# Overall user satisfaction with family planning services and associated quality care factors: a cross-sectional analysis

**DOI:** 10.1186/s12978-018-0615-3

**Published:** 2018-10-12

**Authors:** Allison Marie Slater, Fatima Estrada, Leticia Suarez-Lopez, Elvia de la Vara-Salazar, Lourdes Campero

**Affiliations:** 1 UC Irvine School of Medicine, Irvine, USA; 2 Cátedras CONACYT- Instituto Nacional de Salud Pública, 7a. Cerrada de Fray Pedro de Gante #50, Col. Sección XVI, Tlalpan, C.P. 14080 Ciudad de México, Mexico; 3 Centro de Investigación en Salud Poblacional, Instituto Nacional de Salud Pública, Cuernavaca, Mexico

**Keywords:** Family planning services, Quality health services, User satisfaction, Servicios de planificación familiar, Calidad de los servicios de salud, Satisfacción de los usuarios

## Abstract

**Background:**

Studies of user satisfaction with family planning services (FPSs) have been conducted in different countries, and have been employed to identify ways of improving health, reducing costs and implementing reforms. The present work is the first-ever study undertaken in Mexico on the subject. Our objective was to identify how overall user satisfaction with FPSs in Mexico was related to: healthcare logistics, the functional value of services and the quality of interpersonal relations.

**Methods:** Users of 18 public clinics were surveyed in 2015. Data collected referred to their past and present use of FPSs, as well as to their perceptions of the services provided. We built a logistic regression model with potentially influential variables in order to assess their association with overall satisfaction.

**Results:**

According to the self-reports of the 722 users interviewed, the following factors were decisive in their overall satisfaction with services: receiving sufficient information during visits (OR = 3.38; 95% CI:1.88–6.06), feeling that their opinions were taken into consideration by clinic staff (OR = 2.58; 95% CI:1.14–5.85), feeling that the motives for their visits were addressed (OR = 2.71; 95% CI:1.29–5.71), being assigned enough time for consultation (OR = 2.35; 95% CI:1.26–4.37), having the opportunity to ask questions and clarify doubts (OR = 2.31; 95% CI:1.21–4.43), experiencing no or few interruptions during their medical consultations (OR = 1.97;95% CI:1.10–3.51), and feeling satisfied with the contraceptive method provided (OR = 1.79; 95% CI:1.03–3.11).

**Conclusions:**

Service providers must be kept well informed on the perspective of users concerning user expectations. Taking into account the cultural context and perceived needs of users while providing service would improve the quality of care and, hence, the overall satisfaction of users.

## Resumen

**Antecedentes:**

Los estudios que se han realizado sobre satisfacción de usuarios con servicios de planificación familiar han servido para mejorar los mismos, reducir costos e implementar reformas. El presente análisis tiene como objetivo identificar la asociación de la satisfacción general de los usuarios con cuestiones de logística, valores funcionales y calidad de las relaciones interpersonales.

**Métodos:**

Se encuestaron a 722 usuarios de servicios de planificación familiar en 18 clínicas públicas, en tres entidades federativas de México, durante 2015. La información recabada fue sobre el uso presente y pasado de los servicios de planificación familiar y la percepción sobre la calidad del servicio. Se incluyeron potenciales variables asociadas con la satisfacción general en un modelo de regresión logística.

**Resultados:**

Los factores que resultaron asociados con la satisfacción general del servicio fueron: recibir información adecuada durante la consulta (RM = 3.38; 95% IC:1.88–6.06), sentir que su opinión era tomada en cuenta por el personal de salud (RM =2.58; 95% IC:1.14–5.85), sentir que el motivo de la consulta era atendido (RM =2.71; 95% IC:1.29–5.71), asignar tiempo suficiente a la consulta (RM =2.35; 95% IC:1.26–4.37), tener oportunidad de hacer preguntas y aclarar dudas (RM =2.31; 95% IC:1.21–4.43), tener pocas o ninguna interrupción durante la consulta (RM =1.97;95% IC:1.10–3.51), y sentirse satisfecha con el método anticonceptivo que estaba utilizando (RM =1.79; 95% IC:1.03–3.11).

**Conclusiones:**

Los proveedores de servicios deben mantenerse bien informados sobre las expectativas del usuario. Tener en cuenta el contexto cultural y las necesidades percibidas de los usuarios mientras se presta el servicio mejoraría la calidad de la atención y, por lo tanto, la satisfacción general de los usuarios.

## Plain English summary

Understanding the perspective of users is key to achieving clinically effective and responsive healthcare. Satisfied patients are more likely to comply with recommendations, keep follow-up appointments, and remain with their healthcare provider.

Our study sheds light on the factors affecting user satisfaction with family planning services (FPSs). It therefore contributes to increasing contraceptive use and preventing unwanted pregnancies.

A total of 722 users of public FPSs in three Mexican states were surveyed. The results of our analysis showed that the following factors contributed to their overall satisfaction: receiving adequate information during visits, feeling that their opinions were taken into consideration by clinic staff, feeling that the reasons for their visits were addressed, being assigned sufficient time for consultation, having the opportunity to ask questions and clarify doubts, experiencing no/few interruptions during medical consultations, and feeling satisfied with the contraceptive method provided.

Developing trust is essential to achieving successful family planning (FP) counseling. Healthcare providers must be trained to offer clients humanized care where technical aspects are entwined with strong interpersonal relationships.

## Background

Patient satisfaction with health services has become an important measure of clinical success as the perspective of consumers draws the attention of a growing number of studies and plays an increasingly prominent role in many different fields. In healthcare, studies of patient satisfaction have been used to identify ways of improving health, reducing costs and implementing reforms [1]. The perspective of users is of particular relevance in the pursuit of clinically effective and responsive healthcare; that is, care that is respectful of the values, preferences and expressed needs of patients, provides information and education, is accessible, offers emotional support, involves family and friends, ensures continuity, is concerned about the physical comfort of clients, and delivers services in a logistically coordinated manner [2].

With regard to FPSs specifically, the delivery of clinically effective and responsive care is essential to increasing contraceptive use. This involves treating clients in a respectful, safe and trustworthy manner; making sure that they are satisfied with the contraceptive products supplied; and offering them comprehensive education and counseling [3, 4]. According to a FPS quality framework developed in 1990 by Bruce and still used nowadays, care providers need to combine technical competency with strong interpersonal relations. Moreover, the focus of care must shift from demographic considerations to a client-centered and reproductive-rights approach [5, 6]. Turning their attention to the perspectives of users allows FP counselors not only to identify the aspects of care that matter most to their clients, but also to report opportunities for improving service.

User satisfaction with FPSs has been measured many times in many countries [7, 8]. Some studies have analyzed the relationship among satisfaction, clinical outcomes, and external factors pertaining to the health system, [9] while others have compared the indicators for different healthcare areas [10]. At any rate, much of the existing literature focuses exclusively on female users [5, 11–15]. In Mexico, studies have identified the need to raise the quality of the information offered to users, ensure that the demand for contraceptive methods is met, and intensify outreach efforts with a view to achieving an impact on the adolescent and male populations [16–19]. Several epidemiological studies have compared choices of contraceptive methods, and explored usage of and demand for FPSs by age [20–23]. However, no studies in Mexico have addressed the perspective of users in order to understand the factors associated with their satisfaction. Given the dearth of information on the subject, we undertook to identify how overall user satisfaction with FPSs in Mexico was related to: healthcare logistics, the functional value of services and the quality of interpersonal relations.

## Methods

Our study consisted of a secondary analysis of cross-sectional data. We visited 18 facilities across the states of Morelos, Puebla, and Queretaro, and three public health institutions: the Ministry of Health, the Social Security Institute (*IMSS* by its Spanish initials), and the *IMSS-Prospera* Program. The first provides subsidized healthcare coverage for the population at large, regardless of employment status, thus bearing particular relevance for marginalized communities. The second provides healthcare coverage for the employees and retirees of non-governmental institutions. Finally, the *IMSS-Prospera* welfare program serves populations living in extreme poverty. Data were collected at clinics operated by these institutions in the urban and rural areas of the states mentioned above.

To gather information about users, we designed an instrument based on the indicators of the Quick Investigation of Quality (QIQ) model for clinic-based FP programs [5]. The following general topics were explored: reproductive history, past and present use of FPSs, and factors related with quality of care [6, 24]. We also included indicators for the accessibility, follow-up and continuity of care [5].

Our study was approved by the Research Ethics Committee of the National Institute of Public Health in Mexico. Verbal informed consent was obtained from all participants prior to their interviews. Users were approached at clinic waiting rooms and evaluated for eligibility as participants according to the following criteria and quotas: (1) non-pregnant women of reproductive age in the following age groups: 20–45 years and 15–19 years (70% and 20% of the total sample, respectively), and men in the following age groups: adults and adolescents < 19 years old (at least 10% of the total sample); (2) users who had attended the clinic previously, or first-time users who had completed their first appointment; and (3) users who had not permanently altered their fertility (through tubal ligation, hysterectomy or vasectomy).

## Analysis

The primary outcome analyzed was overall satisfaction with FPSs as reported by users. It was dichotomized as follows: 1 = satisfied and 0 = not/sometimes satisfied. We hypothesized that the key independent variables associated with overall user satisfaction included the following healthcare measures: (1) logistics (adequate wait time, sufficient consultation time, being attended to during visits, and availability of the preferred contraceptive method; (2) functional value (availability of the preferred contraceptive method, satisfaction with the current contraceptive method, provision of adequate information, and having the motives for the visits addressed; and (3) quality of interpersonal relations (having the opportunity to ask questions and clarify doubts during consultations, having no or few interruptions during consultations, being treated respectfully and kindly, enjoying eye contact with staff, having their opinions taken into account, not feeling judged by staff, and having a favorable perception of the medical care received). Our control variables included individual and household characteristics. The following individual variables were considered: sex, age, marital status, years of formal education, reproductive history (prior pregnancies), and length of time attending care at the clinic. The household characteristics which we considered could influence overall satisfaction: residential state and socioeconomic status. Table 1 illustrates how variables were operationalized.

**Table 1:** Description of Variables

Dependent variable	Operationalization
Overall satisfaction with family planning services	Satisfied/Not or sometimes satisfied
Independent variables	
*Measure: healthcare logistics*	
Wait time	< 30 min/30–60 min/ > 60 min
Was offered sufficient consultation time	Yes/No
Was not attended to during visit	Has occurred/Has not occurred
Did not receive contraceptive method because is unavailable	Has occurred/Has not occurred
*Measure: functional value of services*	
Did not receive preferred contraceptive method	Has occurred/Has not occurred
Level of satisfaction with current contraceptive method (acquired at clinic)	Not or slightly satisfied/Fairly or very satisfied
The motive for my visit was addressed	Yes/No
*Measure: quality of interpersonal relations*	
Was given the opportunity to ask questions and clarify doubts during consultation	Yes/No
Received sufficient information	Has occurred/Has not occurred
Was interrupted during consultation	Occurred a lot/Occurred a few times or did not occur
Perceived that medical care received was poor	Has occurred/Has not occurred
Was treated respectfully by staff	Yes/No
Was treated kindly by staff	Yes/No
Enjoyed eye contact with staff	Yes/No
My opinions were taken into consideration	Has occurred/Has not occurred
Felt judged by staff	Has occurred/Has not occurred
Control variables	
Residential state	Morelos/ Puebla/ Queretaro
Socioeconomic status	Low/Medium/High
Sex	Male/Female
Age	< 20 years/20–35 years/ > 35 years
Marital status	Married or in a formal relationship/ Divorced-widowed-single
Years of formal education	< 6/6–9/ > 9 years
Prior pregnancies	0/1/≥2
Length of time attending care at the clinic	< 1 year/1–3 years/> 3 years

We operationalized a variable for socioeconomic status based on a previously established index [25]. We used descriptive statistics and bivariate tests (χ^2^ and Student’s *t* tests) to examine differences in outcomes and covariates between women and men.

We used logistic regression to calculate crude and adjusted odds ratios, modeling the strength of influence that the independent variables had on reported overall satisfaction. For all analyses, we considered a *p*-value ≤0.05 as statistically significant. We included all the variables in a logistic regression model, and confirmed the appropriateness of our specifications through the Hosmer-Lemeshow goodness of fit test. We used StataSE v.12 for analysis.

## Results

### Socio-demographic information

Of the 722 FPS users surveyed, 626 (86.7%) were female and 96 (13.3%) male, with the majority (37.9%) residing in Puebla. Most users exhibited medium socioeconomic status (52.6%), were of reproductive age (between ages 20–35), and had 6–9 years of formal education. Adolescents made up 11.8% (*n* = 85) and adults > 35 years 25.8% (*n* = 186) of the sample. Approximately two-thirds of users were living in union, and a large majority (91.0%) had been pregnant or had had their partner become pregnant at least once (Table 2).

**Table 2:** Socio-demographic information of surveyed users

	Men		Women		All	
Residential state	*n* (%)		*n* (%)		*N* (%)	
Morelos	27	28.12%	197	31.47%	224	31.02%
Puebla	31	32.39%	243	38.82%	274	37.95%
Queretaro	38	39.58%	186	29.71%	224	31.02%
Socioeconomic status (SES)*						
Low SES	12	12.50%	173	27.64%	185	25.62%
Medium SES	52	54.17%	328	52.40%	380	52.63%
High SES	32	33.33%	125	19.97%	157	21.75%
Healthcare institution						
Ministry of Health	27	28.12%	245	39.14%	272	37.67%
*IMSS*	47	48.96%	198	31.63%	245	33.93%
*IMSS-Prospera*	22	22.92%	183	29.23%	205	28.39%
Age						
< 20 years old	17	17.71%	68	10.86%	85	11.77%
20–24	26	27.08%	130	20.77%	156	21.61%
25–29	19	19.79%	142	22.68%	161	22.30%
30–34	11	11.46%	123	19.65%	134	18.56%
≥ 35	23	23.96%	163	36.04%	186	25.76%
Years of formal education						
< 6 years	6	6.25%	64	10.22%	70	9.70%
6–9 years	68	70.83%	417	66.61%	485	67.17%
> 9 years	22	22.92%	144	23.00%	166	22.99%
Marital status						
Married or in union	65	67.71%	526	84.16%	591	81.97%
Divorced, widowed or single	31	32.29%	99	15.84%	130	18.03%
Reproductive history	# obs	Mean ± SD	# obs	Mean ± SD	# obs	Mean ± SD
Number of pregnancies*	67	2.30 ± 1.50	589	2.41 ± 1.39	656	2.40 ± 1.40
Age at time of first pregnancy*	65	21.91 ± 4.73	590	19.68 ± 4.00	655	19.90 ± 4.13
Number of liveborns	63	2.22 ± 1.53	587	2.20 ± 1.22	650	2.20 ± 1.25
Age at time of last pregnancy*	63	27.21 ± 6.58	577	25.03 ± 5.92	640	25.24 ± 6.01

*p ≤ 0.05

When comparing user characteristics by sex**,** we found significant differences regarding SES, number of prior pregnancies, and ages at the time of the first and most recent pregnancies, but not with respect to the key independent variables. More women than men had low SES (27.64% of women as opposed to 12.50% of men), and on average, women reported having had more pregnancies than did the partners of their male counterparts. Women were also younger on average at the time of their first pregnancy than were male users when their partners experienced their first pregnancy (Table 1).

### User satisfaction with FPSs

Among all those surveyed (*n* = 722), nearly one-fifth (18.7%) reported being dissatisfied with FPSs. According to the results adjusted by state, socioeconomic status, sex, age, marital status, education, prior pregnancies and length of time the user had attended care at the clinic, the most important factors contributing to overall satisfaction were being given adequate information (OR 3.38; 95% CI: 1.88–6.06); feeling that the motive for their visit was addressed (OR 2.71; 95% CI: 1.29–5.71); and feeling that their opinions had been taken into consideration by clinic staff (OR 2.58; 95% CI: 1.14–5.85).

Other significant factors included being provided sufficient time for consultation (OR 2.35; 95% CI: 1.26–4.37), having the opportunity to ask questions and clarify doubts (OR 2.31; 95% CI: 1.21–4.43), waiting less than 30 min for service (OR 2.10; 95% CI: 1.01–4.34), experiencing few interruptions by staff during consultation (OR 1.97; 95% CI: 1.10–3.51), and being satisfied with the contraceptive method provided (OR 1.79; 95% CI: 1.03–3.11) (Table 3).

**Table 3:** Logistic regression model: crude and adjusted odds ratios for factors affecting overall user satisfaction

	Odds ratio crude (95% CI)	Odds ratio adjusted (95% CI)
Residential state		
Morelos	1.00	1.00
Puebla	1.43 (0.90–2.26)	1.19 (0.61–2.32)
Queretaro	0.97 (0.62–1.54)	0.96 (0.49–1.89)
Socioeconomic status		
Low	1.00	1.00
Medium	0.98 (0.61–1.56)	1.12 (0.55–2.26)
High	0.61 (0.36–1.03)	0.97 (0.40–2.33)
Sex		
Male	1.00	1.00
Female	1.00 (0.58–1.74)	0.89 (0.40–1.97)
Age group		
< 20 years	1.00	1.00
20–34	0.83 (0.46–1.53)	0.38 (0.15–1.01)
> 35	1.21 (0.61–2.40)	0.50 (0.16–1.60)
Marital status		
Married or living in union	1.00	1.00
Divorced, widowed or single	1.47 (0.93–2.31)	1.09 (0.51–2.32)
Years of formal education		
< 6 years	1.00	1.00
6–9 years	0.78 (0.39–1.59)	1.50 (0.51–4.32)
> 9 years	0.50 (0.23–1.06)	0.88 (0.26–2.96)
Prior pregnancies		
0	1.00	1.00
1	1.03 (0.53–2.01)	3.06 (1.01–9.27)
≥ 2	1.52 (0.81–2.84)	4.60 (1.42–14.93)
Length of time attending care at the clinic		
< 1 year	1.00	1.00
1–3	1.50 (0.88–2.56)	1.54 (0.71–3.33)
> 3	1.00 (0.65–1.55)	1.08 (0.54–2.16)
Wait time		
> 60 min	1.00	1.00
30–60 min	2.29 (1.41–3.72)	0.85 (0.44–1.64)
< 30 min	4.39 (2.61–7.38)	2.10 (1.01–4.34)
Was offered sufficient consultation time		
No	1.00	1.00
Yes	8.08 (5.31–12.31)	2.35 (1.26–4.37)
Was not attended to during visit		
Has occurred	1.00	1.00
Has not occurred	5.33 (3.44–8.27)	1.13 (0.54–2.39)
Did not receive contraceptive method because was unavailable		
Has occurred	1.00	1.00
Has not occurred	1.96 (1.26–3.03)	1.21 (0.63–2.33)
Did not receive preferred contraceptive method		
Has occurred	1.00	1.00
Has not occurred	5.87 (2.87–11.97)	1.78 (0.62–5.11)
Level of satisfaction with current contraceptive method (acquired at clinic)		
Not or slightly satisfied	1.00	1.00
Fairly or very satisfied	3.54 (2.02–6.16)	1.79 (1.03–3.11)
The motive for the visit was addressed		
No	1.00	1.00
Yes	11.38 (6.78–19.07)	2.71 (1.29–5.71)
Was given the opportunity to ask questions and clarify doubts during consultation		
No	1.00	1.00
Yes	10.54 (6.69–16.61)	2.31 (1.21–4.43)
Received sufficient information		
Has occurred.	1.00	1.00
Has not occurred.	4.62 (3.12–6.84)	3.38 (1.88–6.06)
Was interrupted during consultation		
Occurred a lot	1.00	1.00
Occurred a few times or did not occur	4.79 (3.23–7.12)	1.97 (1.10–3.51)
Perceived that medical care received was poor		
Has occurred	1.00	1.00
Has not occurred	7.75 (5.02–11.95)	1.16 (0.51–2.66)
Was treated respectfully by staff		
No	1.00	1.00
Yes	11.82 (7.01–19.91)	2.18 (0.73–6.50)
Was treated kindly by staff		
No	1.00	1.00
Yes	10.94 (6.86–17.46)	1.65 (0.60–4.56)
Enjoyed eye contact during conversations with staff		
No	1.00	1.00
Yes	5.27 (3.51–7.91)	1.40 (0.75–2.62)
The users´ opinions were taken into consideration		
Has occurred	1.00	1.00
Has not occurred	8.01 (4.96–12.91)	2.58 (1.14–5.85)
Felt judged by staff		
Has occurred	1.00	1.00
Has not occurred	5.95 (3.43–10.34)	0.98 (0.38–2.53)

Additionally, having previously been pregnant or having had a partner become pregnant contributed significantly to increased satisfaction (OR 3.06; 95% CI: 1.01–9.27 for one prior pregnancy, and OR 4.60; 95% CI: 1.42–14.93 for more than two prior pregnancies) (Table 3). The other variables in the regression model did not contribute significantly to increased satisfaction, though the complete regression model explains 40% of the variance in user satisfaction (pseudo R^2^ = 0.40).

## Discussion

We sought to study how healthcare factors related to logistics, functional value and quality of interpersonal relations contributed to overall user satisfaction with family planning services (FPSs). The finding that approximately 80% of users reported being generally satisfied with the services provided by their clinics is in line with other studies [26–29].

Less is known about what clients perceive as quality care. A recent systematic review shows that clients were most likely to identify issues related to accessibility, client-centeredness and, to a lesser extent, equitability as key elements. Efficiency and effectiveness in care were less important. It is worth noting, however, that the majority of studies in the review were conducted in the USA [26]. Our analysis indicated that issues related to efficiency (logistics) and effectiveness (functional values) were associated with overall satisfaction, while issues related to client-centeredness (interpersonal relations) were less important.

Our results are similar to those found in other Latin American countries, where patients have reported being highly dissatisfied particularly as regards the information offered by care providers on contraceptive methods, and the lack of opportunity to ask questions and clarify doubts. These results suggest that these factors are particularly important and shape the overall impression of services more strongly in Latin American populations than in developed countries [5].

Key results include the prominent role that reproductive history plays in contributing to higher satisfaction; notably, the highest odds ratio in our model was associated with having had more than two prior pregnancies. This lends credence to the theory that the satisfaction of users is strongly related to their expectations [24]. Simply having utilized a contraceptive method when seeking to limit fertility represents a met expectation. Other indicators such as having the motives for visits addressed and being satisfied with the contraceptive methods provided also reinforce the hypothesis that meeting client expectations is a primary factor in their evaluation of services [27].

We found that the reproductive age group (20–35 years) reported significantly lower levels of satisfaction than the adolescent and > 35 groups. Socio-demographic factors that have previously been shown to influence patient satisfaction with health services include increased age and better health, although the literature is not conclusive and may not be applicable to outpatient FPSs; [28] one study found no association between age and reported satisfaction [12]. We hypothesize that the decreased level of overall satisfaction with FPSs in this age group may be attributable to the higher expectations of its members due to a higher level of experience with FPSs and contraceptive methods. This result suggests that tailoring services to address specific client characteristics and needs is an important component in providing quality FPSs.

We did not find any statistically significant differences between men and women in the key independent variables analyzed. One reason could be the small number of men receiving health services in general, and reproductive health services in particular. This evidence suggests that health services have not created effective strategies for incorporating men into the system, [30] leaving the responsibility for fertility control with women [31, 32]. It is necessary to recognize men as enjoying reproductive rights and adapt FPSs to their needs [31].

While our study provides important insights, some limitations should be noted. One of them is the potential for courtesy bias, as surveys were administered within the clinics. In addition, users of public FPSs in Mexico may not have the resources that would allow them to exercise choice over the type and location of clinic they are using, particularly those associated with the Ministry of Health. Users of these clinics may have such low expectations that even if the service is poor, they evaluate it positively because it exceeds their expectations [28].

Although this study included both male and female users of FPSs, it is necessary to increase the sample size of men in order to obtain a more complete perspective on the differences between the sexes. Other key factors that could affect overall satisfaction, such as technical issues, are not included in this study; measuring them is essential for providing a deeper understanding of this topic.

It would be useful to conduct loosely structured interviews outside the clinic to obtain a more nuanced picture of client experiences at the clinic and more detailed opinions of quality and satisfaction. Additionally, broadening data collection to include the private sector would serve to complement the information in this study pertaining to the public sector, the principal provider of FP methods in Mexico.

## Conclusions

This is one of few large, cross-sectional studies to take user satisfaction into account as a component of success in the implementation of FP programs in the public sector. Our results offer insights regarding how FPSs could be improved by providing more respectful user care, particularly by supplying adequate information and taking the opinions of clients into consideration. The latter two have been shown to impact overall satisfaction markedly and could prove an important pathway to increasing use of FPSs.

Establishing trust is an essential part of the FPS counseling process. If users feel comfortable, they will be able to express their needs and doubts more openly. Health providers must be sensitized and trained to offer humanized counseling, strengthening both the technical and the interpersonal aspects of their relationship with users. It is necessary to implement FP policies taking the cultural context and the needs of users into consideration as a way of enhancing the quality of service.

## Abbreviations

CI: Confidence interval
FP: Family planning
FPSs: Family Planning Services
IMSS: Instituto Mexicano del Seguro Social
OR: Odds ratio
QIQ: Quick Investigation of Quality
SES: Socioeconomic status
